# Does sexual behaviour of people with HIV reflect antiretroviral therapy as a preventive strategy? A cross-sectional study among outpatients in Kenya

**DOI:** 10.1186/s12889-019-7581-8

**Published:** 2019-09-11

**Authors:** Kennedy Nkhoma, Aabid Ahmed, Zipporah Alli, Lorraine Sherr, Richard Harding

**Affiliations:** 1 Faculty of Nursing, Midwifery and Palliative Care, Cicely Saunders Institute of Palliative Care Policy and Rehabilitation, Denmark Hill Campus, King’s College London, Bessemer Road, London, SE5 9PJ UK; 2 Bomu Hospital, Mombasa, Kenya; 3 Kenya Hospices and Palliative Care Association, Nairobi, Kenya; 4 University College London, London, UK

**Keywords:** Sexual risk taking, HIV/AIDS, Antiretroviral therapy

## Abstract

**Background:**

The World Health Organisation (WHO) advocates early initiation of HIV treatment as a prevention strategy among people living with HIV. There is strong evidence for the effectiveness of antiretroviral therapy (ART) as a preventive tool for HIV transmission. We aimed to determine the sexual behaviour of HIV outpatients and assess if it reflects the current preventive strategy for HIV transmission.

**Methods:**

We conducted a cross-sectional study among adult (aged at least 18 years) patients with confirmed HIV diagnosis, and aware of their diagnosis, attending HIV outpatient care in Kenya. Data were gathered through self-report (using validated questionnaires) and file extraction. Multivariate logistic regression assessed the association between sexual risk taking behaviour controlling for gender, HIV clinical stage, HIV treatment status, Tuberculosis (TB) treatment status, and CD4 count.

**Results:**

We recruited *n* = 400 participants (*n* = 280[70%] female gender). The mean age was 39.4 (SD = 9.9) years. The mean CD4 count was 393.7 (SD = 238.2) and ranged from 2 to 1470 cells/mm^3^. *N* = 61 (15.64%) were on TB treatment. The majority (*n* = 366, 91.5%) were on ART. Just over half (*n* = 202, 50.5%) reported having a sexual partner. Of these *n* = 33 (16.1%) reported having unprotected sexual intercourse with a person of unknown HIV status in the previous 3 months.

Multivariate analysis showed that participants not on ART (HIV treatment) were more likely to report unprotected sexual intercourse compared to those who were on ART (odds ratio .25, 95% CI .09 to .69; *P* = 0.007).

Participants at early stage of HIV infection (stages 1/2) were more likely to report unprotected sexual intercourse compared to participants at advanced HIV infection (stages 3/4) (odds ratio .34, 95% CI .13 to .92; *P* = 0.035). Males participants were more likely to be involved in sexual risk taking behaviours compared to female participants (odds ratio .36, 95% CI .16 to .82; *P* = 0.015). TB treatment status, and CD4 count were not significantly associated with sexual risk taking.

**Conclusion:**

Participants not on ART have more unprotected sexual intercourse than those who are on ART. This calls for the need to scale up coverage and early ART initiation in order to reduce transmission of HIV.

## Background

Kenya has one of the highest prevalence and burden of HIV infection. In terms of HIV epidemic, Kenya seats on fourth position alongside Mozambique and Uganda. At the end of 2017, there were 1.5 million people living with HIV in Kenya with 4.8% adult prevalence [1, 2].

This continued high prevalence of HIV in Kenya among adults are likely to be driven by a range of sexual behaviours such as inconsistent condom use and multiple sexual partners [1]. There have been inconsistent reports about sexual behaviour of people living with HIV (PLWH). Some studies report that those on antiretroviral therapy (ART) are more likely to engage in unprotected sexual intercourse, for instance studies involving the youth [3, 4] women [5], gay men [6, 7] and adults [8, 9], while other studies report that those on ART are less likely to engage in unprotected sex [10–13]. An estimated 30% of new HIV transmissions are among people from key populations such as gay men and people who inject drugs [14]. Among people who inject drugs 18.3% are HIV positive [14]. Early initiation of ART has been reported to reduce HIV transmission among discordant couples. A trial among predominantly heterosexual discordant couples reported 96% reduction of risk of HIV transmission [15]. A recent prospective observational study among discordant couples reported zero within couple transmission of HIV after 13 months follow-up [16]. Systematic reviews of randomised controlled trials (RCT) and observational studies also report that ART reduces HIV transmission among discordant couples due to undetectable viral loads [17, 18].

There has been some significant progress in terms of implementing the 90–90-90 UNAIDS policy in Kenya – which aims to ensure that 90% for of those with HIV know their status, that 90% of those with HIV are on treatment and 90% of those on treatment will be virally suppressed. At the end of 2018, 75% of the population who are HIV positive started ART (HIV treatment). Furthermore there has been a decline in mortality from 51,000 in 2010 to 36,000 in 2015 due to increased uptake of ART [19, 20].

Potent HIV prevention strategies are required to ensure there is no HIV transmission from infected persons to uninfected partners.

We therefore conducted a cross-sectional study among HIV patients to determine if their sexual behaviour reflects a new understanding of ART as a prevention strategy for reducing the spread of HIV.

## Methods

Informed by the health belief model, which attempts to explain and predict health behaviours among individuals [21], we carried out a cross-sectional study using self-report and supplemented this with clinic data drawn from file extraction. We recruited adults, at least 18 years old, HIV positive and aware of their status. Participants were attending HIV outpatient care at Bomu hospital in Mombasa, Kenya. We excluded patients younger than 18 years of age, and those not aware of their diagnosis, or those unable to give self-report data with assistance from the researcher due to significant physical or psychological impairment. Kenyan Medical Research Institute provided ethics approval for this study.

### Procedures

The details of the study procedures have been explained in our previous publications [22, 23]. We employed a research assistant who implemented all research activities at the study site.

### Data collection

The following variables were extracted from patient’s records by the researcher: CD_4_, HIV treatment status and TB treatment status. We planned to extract data on viral load, but these data were not available for the majority of the participants. The other data were self-report and the researcher used a private room at the HIV clinic to collect this data. The researcher recorded responses as participants gave answers verbally. This procedure ensured that all the questionnaires were completed by the researcher, thereby reducing any potential response bias which may occur by mixing self-completion and researcher-completion data. We used the following tools to gather data, which have previously been used in sub-Saharan Africa among people with HIV:

1. Sexual behaviour, and adherence questionnaire: Sexual behaviour section consists of two items: (1) sexual partners in the last 3 months, and (2) unprotected sexual intercourse with a person of unknown HIV status in the previous 3 months. The adherence questionnaire asks participants to record the number of doses they have missed in the past 7 days [24, 25]. This questionnaire was recently used in a clinical trial conducted in Kenya among HIV patients [26, 27]. Questions on HIV risk behaviour were asked face-to-face by a researcher who read the questions aloud to the participants and thereafter recorded self-reported responses.
2. Demographics, and socioeconomic status: Demographics and socioeconomics data were gatherer using the Demographic and Health Survey (DHS) questionnaire [28]. This consists of variables such as house construction, possession of items, fuel supply, and water source. These variables were used to calculate the wealth of the participants. The wealth quintile variable was created in line with the methods of DHS [28]. We used factor analysis with principal component analysis to create a continuous variable which was then converted into quintiles. DHS has previously been used in HIV research in sub-Saharan Africa [27, 29].

### Sample size calculation

We calculated our sample size based on HIV prevalence in Kenya. We have provided these details in our previous publications [22, 23].

### Data analysis

We used Stata version 14 to analyse the data [30]. Descriptive statistics were used to profile the demographic, socioeconomic and clinical characteristics of participants. WHO clinical stages were combined (1 and 2 combined to one group, likewise 3 and 4, because of small numbers).

A series of logistic regression analysis were conducted. The dependent outcome was sexual risk taking, i.e. penetrative sexual intercourse without a condom in the previous 3 months with a person that the participant was not sure also had HIV (binary variable: yes/no). Covariates were demographic variables (age, gender, education, and wealth in quintiles), clinical variables (WHO clinical stage: 1/2 vs 3/4, CD4 count in quintiles, TB treatment: yes/no, ART treatment: yes/no). Initially we conducted a univariate analysis for demographic, clinical variables and outcome variable. We then conducted adjusted/multivariate analysis for demographic variables and outcome variable falling within less than 25% *p* value [31]. Clinical variables (HIV treatment status, CD4 count, WHO clinical stage, TB treatment status) were forced into the multivariate model regardless on the outcome of the univariate.

This is because these are confounding variables frequently associated with HIV risk behaviours. We were interested in risk factor modelling not just prediction. Studies have shown that researchers can return confounding variables as long as sample sizes are larger because correct retention increases with sample size [32].

Sexual behaviour is frequently associated with clinical variables such as treatment status [7]. All cases with missing data were excluded from the models.

## Results

Demographic characteristics of the participants are presented in Table 1. We approached *n* = 475, of these *n* = 400 consented and were recruited, with a response rate of 84.2%. The mean age of the participants was 39.4 (SD 9.9) years. Most of the participants (*n* = 280, 70%) were females. Just over half of our participants (*n* = 213, 53.25%) attended primary school, and just over a quarter of the our participants (*n* = 113, 28.25%) went to secondary school.

**Table 1:** Demographic and clinical characteristics (*N* = 400)

Demographic characteristics	
Gender	
Male	120 (30%)
Female	280 (70%)
Mean age (SD)	39.4 (9.91)
Relationship^a^	
In a relationship	209 (52.38%)
Not in relationship	190 (47.62%)
Education	
One or two years	45 (11.25%)
Primary or > 2 years	213 (53.25%)
Secondary	113 (28.25%)
Diploma	24 (6%)
Degree or higher	5 (1.25%)
Wealth quintile	
Poorest	101 (25.25%)
Middle poor	59 (14.75%)
Middle	80 (20%)
Middle wealthy	80 (20%)
Wealthiest	80 (20%)
Behavioural characteristics	
Told someone HIV Status	
Yes	373 (93.25%)
No	27 (6.75%)
Missed doses^b^	
Yes	64 (17.49%)
No	302 (82.51%)
Mean number of missed doses	0.36 (1.28%)
Range (number of missed doses)	1–14
Missed dose and unprotected sexual intercourse	
Missed 1 or more doses but did not have unprotected sex	31 (91.18%)
Missed 1 or more doses and had unprotected sex	3 (8.82%)
Missed 1 or more doses and number of sexual partners	
No sexual partner	32 (50.00%)
One sexual partner	32 (50.00%)
Sexual partner	
No	198 (49.50%)
One sexual partner	187 (46.75%)
More than one sexual partner	15 (3.75%)
Unprotected sexual intercourse with someone of unknown HIV status^c^	
Yes	33 (16.1%)
No	169 (82.44%)
Sexual behaviour	
With men only	264 (66%)
With women only	133 (33.25)
With men and women	3 (0.75%)
Sexual orientation by gender	
Males	
Sex with men only	18 (15%)
Sex with women only	101 (84.17%)
Sex with men and women	1(0.83%)
Females	
Sex with men only	246(87.86%)
Sex with women only	32 (11.43%)
Sex with men and women	2(0.71%)
Unprotected by gender	
Males	17 (51.52)
Females	16 (48.48)
Unprotected sex with men only	19 (57.58)
CD_4_ count	
Mean	393.7 (238.2)
Range	2–1470
WHO Stage	
Stage 1/2	52 (13%)
Stage 3/4	348 (87%)
Patient on ART	
Yes	366 (91.5%)
No	34 (8.5%)
Unprotected sex and ART status	
On ART	25 (75.76)
Not on ART	8 (24.24)
Patient on TB treatment^d^	
Yes	61 (15.64%)
No	329 (84.36%)

^a^1 missing

^b^34 not on ART

^c^198 did not have a sexual partner

^d^10 missing

### Treatment variable and sexual behaviour variables

The majority of the patients were taking ART (*n* = 366, 91.5%), with *n* = 61 (15.64%) on concurrent TB treatment. The majority were on Nucleoside/nucleotide reverse transcriptase inhibitors (NRTIs/NtRTIs) (*n* = 262, 90.50%) and Non-nucleoside reverse transcriptase inhibitors (NNRTIs) (*n* = 319, 79.75%) as presented on Table 2. The mean CD4 count was 393.7 (SD = 238.2) and ranged from 2 to 1470 cells/mm^3^. The majority of our participants had advanced HIV/AIDS infection (stage III/IV) (*n* = 348, 87%). Most of these participants had disclosed their HIV status at least to one person (*n* = 373, 93.25%). Adherence in the preceding 7 days was 75.5% fully adherent (*n* = 302), with 16% reported missing 1 or more doses (*n* = 64). The mean (SD) number of doses missed was 0.36 (SD = 1.28) and ranged from 1 to 14 doses. Of the *n* = 400 participants, just over half *n* = 202 (50.5%) reported having one or more sexual partners in the last 3 months. Of those who reported having a sexual partner in the previous 3 months *n* = 33 (16.1%) reported that they had unprotected sex with a person of unknown HIV status.

**Table 2:** ART Regimen

ART classification	Frequency
Nucleoside/nucleotide reverse transcriptase inhibitors (NRTIs/NtRTIs)	362 (90.50)
Non-nucleoside reverse transcriptase inhibitors (NNRTIs)	319 (79.75)
Protease inhibitors	16 (4.00)
Integrase inhibitors	1 (0.25)

### Univariate analysis

Table 3 shows univariate analysis of the logistic regression model. Sexual risk taking was significantly associated with being on HIV treatment and gender.

**Table 3:** Associations of factors with sexual risk taking (unprotected sexual intercourse with someone of unknown status): Univariate analysis (*n* = 202)

Outcome/dependent variable	Independent variable	Odds ratio (95% CI)	Z score	*P* value
Sexual risk taking (Unprotected sex with someone of unknown status: yes or no)	Age	0.98 (0.95 to 1.03)	−0.54	0.59
	Women vs men (ref female)	0.42 (0.19 to 0.89)	−2.26	0.024*
	WHO stage 1/2 vs 3/4	0.42 (0.17 to 1.06)	−1.85	0.065*
	ART (HIV treatment) (ref yes)	0.28 (0.11 to .74)	−2.57	0.010*
	TB treatment (ref yes)	1.14 (0.43 to 3.03)	0.27	0.79^§^
	CD4 count (in quintile)	1.10 (0.84 to 1.44)	0.68	0.49^§^
	Education	1.07 (0.65 to 1.75)	0.27	0.79
	Wealth (quintile)	1.01 (0.78 to 1.32)	0.09	0.93

*< 25% significance and included in multivariate analysis

^§^Not significant at 25% but forced into the multivariate analysis

Participants who were not on ART (HIV treatment) were likely to report sexual risk-taking behaviour (unprotected sexual intercourse) compared to participants who were on ART (odds ratio .28, 95% CI .11 to .74; *P* = 0.010). Male participants were more likely to be involved in sexual risk-taking behaviours compared to female participants (odds ratio .42, 95% CI .19 to .89; *P* = 0.024). Age, clinical stage, CD4 count, education, wealth were not significantly associated with sexual risk taking.

### Multivariate analysis

When adjustments were made (Table 4) for gender, clinical variables (HIV treatment (yes/no), CD4count (quintiles), TB treatment (yes/no), and HIV clinical stage) in the multivariate analysis, ART (HIV treatment), gender and clinical stage were significantly associated with sexual risk taking.

**Table 4:** Associations of factors with sexual risk taking (unprotected sexual intercourse with someone of unknown HIV status): Multivariate analysis (*n* = 202)

Outcome/dependent variable	Independent variable	Odds ratio (95% CI)	Z score	*P* value
Sexual risk taking (Unprotected sex with someone of unknown status: yes or no)	ART (HIV treatment) (ref yes)	0.25 (0.09 to .69)	−2.68	0.007*
	WHO stage 1/2 vs 3/4	0.34 (0.13 to 0.92)	−2.11	0.035*
	Men vs women (ref male)	0.36 (0.16 to 0.82)	−2.43	0.015*
	CD4 (quintiles) (ref very low)	1.18 (0.88 to 1.59)	1.13	0.26
	TB treatment (ref yes)	1.26 (0.45 to 3.52)	0.44	0.66

*statistically significant

Participants who were not on ART were more likely to report unprotected sexual intercourse with someone of unknown HIV status in the previous 3 months compared to participants who were on ART (odds ratio .25, 95% CI .09 to .69; *P* = 0.007).

Participants who were at stages 1/2 were more likely to have unprotected sexual intercourse than participants who were at stages 3/4 of HIV infection (odds ratio .34, 95% CI .13 to .92; *P* = 0.035).

Male participants were more likely to be involved in sexual risk taking behaviour compared to female participants (odds ratio .36, 95% CI .16 to .82; *P* = 0.015).

## Discussion

Results from this study showed that participants who were not on ART were likely to have unprotected sexual intercourse with someone of unknown HIV status in the previous 3 months compared to participants who were on ART. Our results show high risk of HIV transmission among participants not on ART.

This result is an important public health concern because participants were at risk of acquiring other viruses (re-infection with new strains of HIV from people they were having unprotected sexual intercourse with, (assuming they were also HIV positive and not on treatment), furthermore they were at risk of spreading other forms of viruses (onward transmission of HIV) [33, 34]. However this was going to be a different story if these respondents were on ART (HIV treatment) because the risk of transmission is reduced when one is on HIV treatment and viral load has been suppressed [1]. International Business Times (2013) reported that in 2013, condom distribution fell far below demand in Kenya for 2 years because they were not budgeted for [35]. This may well have contributed to reasons why participants practised unprotected sexual intercourse. Moreover there were reports that men were reusing condoms, or were using plastic bags due to shortage of free condom supply in public facilities [36]. On the other hand, condoms may be available but this does not guarantee that they will be used. A demographic health survey reported that only 40% of women and 43% of men with multiple sexual partners used a condom in Kenya [37].

Our results are consistent with recent evidence from a study conducted in South Africa which reported that 25% of the sample awaiting to start ART had unprotected sexual intercourse with HIV negative or untested partners [38, 39]. However previous work from a cross-sectional study conducted in South Africa [40] and meta-analysis of cross-sectional studies conducted in sub-Saharan Africa [41] reported that ART is significantly associated with decreased sexual risk behaviours.

Another important finding from this study is that participants who were at stages 1/2 were more likely to have unprotected sexual intercourse than participants who were at stages 3/4 of HIV infection. This is possibly related to the fact that patients at stage 3/4 of HIV infection are eligible for ART and received health education and information about positive living with HIV which includes practising safe sex, such as using condoms. Our results are in line with a prospective, longitudinal study conducted in Uganda which reported that unprotected sexual intercourse decreased after ART initiation [42]. Another explanation for higher sexual risk taking at stages 1/2 of HIV infection may be due to the fact that at stage 3/4 participants were quite sick and therefore they did not have interest in sexual intercourse. A cross-sectional internet based survey in the USA reported sexual problems among gay men with advanced HIV/AIDS infection [43].

Male participants were more likely to be involved in sexual risk taking behaviour compared to female participants. Men dominate sexual relationships hence practice unsafe sex even when they know the risks associated with this behaviour [19].

### Study strengths

We used questionnaires validated in local population and these have previously been used among people with HIV in a randomised control trial [26, 27]. An experienced researcher administered the questionnaires face-to-face with study participants. All questions were read aloud to the participants and thereafter responses were recorded. This helped to reduce cases with missing data. Furthermore, we included broad relevant clinical data on patient reported outcomes. Our study had a response rate of 84.2%.

### Limitations

This data did not have viral load measures, one of the important variable which we could have used to understand if the unprotected sex was associated with risk of HIV transmission. We did not ask participants about sexual assault, forced sexual intercourse, alcohol and drug use and other risk factors that may predispose the transmission of HIV. These factors are important in assessing the risk of HIV transmission and infection. These data were cross-sectional therefore we cannot determine causation, but associations. We do not know if participants who had unprotected sexual intercourse were having sexual encounter with HIV negative partners which means they were at risk of transmitting HIV. A follow-up study to trace sexual partners of study participants could have helped to screen them for HIV and then offer appropriate care depending on the outcome of HIV test.

## Conclusions

Kenya has to effectively implement the 90–90-90 UNAIDS policy in order to mitigate the impact of HIV in the country. It is interesting to note that in 2015, Kenya adopted the WHO recommendations to immediately offer treatment to people diagnosed with HIV. It is also interesting that in 2017 Kenya rolled-out PrEP for key populations such as injecting drug users, sero-discordant couples, commercial sex workers, and those with history of recent sexually transmitted infection. Recent UNAIDS (2018) global report shows that 75% of those living with HIV are on ART, and 63% of all people living with HIV are virally suppressed [2]. Kenya has a long way to achieve the 90–90-90 policy by 2020. The country has to improve on scaling up ART so that all people who test positive should be put on ART.

Viral load testing needs to be conducted routinely in order to assess the effectiveness of ART and effectively monitor the 90–90-90 policy. Health education and information provision to the community on condom use to prevent the transmission of HIV infection among those not on ART treatment needs to continue and be strengthened.

## Data Availability

The datasets used and/or analysed during the current study are available from the corresponding author on reasonable request.

## Abbreviations

AIDS: Acquired immune deficiency syndrome
ART: Antiretroviral therapy
DHS: Demographic and Health Survey
HIV: Human immune deficiency virus
RCT: Randomised Controlled Trial
TB: Tuberculosis
UNAIDS: Joint United Nations Programme on HIV/AIDS
WHO: World Health Organisation
